# The Evolution of Cardiovascular Surgery in Elderly Patient: A Review of Current Options and Outcomes

**DOI:** 10.1155/2014/736298

**Published:** 2014-04-10

**Authors:** Francesco Nicolini, Andrea Agostinelli, Antonella Vezzani, Tullio Manca, Filippo Benassi, Alberto Molardi, Tiziano Gherli

**Affiliations:** ^1^Unità di Cardiochirurgia, Dipartimento di Medicina Clinica e Sperimentale, Università degli Studi di Parma, Via A. Gramsci 14, 43126 Parma, Italy; ^2^Unità Operativa di Cardiochirurgia, Dipartimento Cardio-Nefro-Polmonare, Azienda Ospedaliero-Universitaria di Parma, Via A. Gramsci 14, 43126 Parma, Italy

## Abstract

Due to the increase in average life expectancy and the higher incidence of cardiovascular disease with advancing age, more elderly patients present for cardiac surgery nowadays. Advances in pre- and postoperative care have led to the possibility that an increasing number of elderly patients can be operated on safely and with a satisfactory outcome. Currently, coronary artery bypass surgery, aortic and mitral valve surgery, and major surgery of the aorta are performed in elderly patients. The data available show that most cardiac surgical procedures can be performed in elderly patients with a satisfactory outcome. Nevertheless, the risk for these patients is only acceptable in the absence of comorbidities. In particular, renal dysfunction, cerebrovascular disease, and poor clinical state are associated with a worse outcome in elderly patients. Careful patient selection, flawless surgery, meticulous hemostasis, perfect anesthesia, and adequate myocardial protection are basic requirements for the success of cardiac surgery in elderly patients. The care of elderly cardiac surgical patients can be improved only through the strict collaboration of geriatricians, anesthesiologists, cardiologists, and cardiac surgeons, in order to obtain a tailored treatment for each individual patient.

## 1. Introduction

Developments in myocardial protection, surgical technique, extracorporeal circulation, and anesthesiological management have led to reduced mortality and morbidity rates in cardiac surgery through the 1990s and early 2000s [1, 2]. Moreover, it is well demonstrated that the median age of global and industrialized countries populations is increasing. Thirteen million US citizens are currently over the age of 75, and this number is rapidly increasing over the next 50 years [3].

Another important issue is that approximately 25% of the population over 75 years suffers from symptoms of cardiovascular diseases [4]; the contemporary aging of society is consequently leading to an increasing proportion of elderly patients who are potential candidates for cardiac surgery. In Germany, it has well documented that the average age of cardiac surgical patients increased from 1990 to 2007 from 55.8 years to 68.8 years and that the proportion of octogenarians is 9.8% [5]. Although there is evidence suggesting improved quality of life in elderly patients who have undergone cardiac surgery [6, 7], a correlation between age and morbidity and mortality risk has been well established [8]. Nevertheless, among patients undergoing cardiac surgery, the trend over time demonstrates an increase in case complexity. In the field of coronary artery disease, elderly patients benefit from surgical revascularization (CABG) more than from conservative treatment [9]. However, the developments in percutaneous coronary interventions (PCI) have resulted in a marked drop in potential candidates for isolated CABG [10] and in a shift in referrals for cardiac surgery away from isolated CABG and towards more complex interventions, sometimes in very old and frail patients. These high-risk patients usually require prolonged ICU stays and are at increased risk for mortality, major morbidity, and prolonged institutional care [11, 12].

An open question concerns the influence of the biological aging process on the operative risk, regardless of the type of surgery performed. It can be assumed that chronological age is a risk factor for morbidity and mortality after cardiac surgery, although the number and severity of comorbidities that usually increase with chronological age are important additional factors for the perioperative and long-term prognosis of these high-risk patients. This aging surgical population, not unexpectedly, has a relatively greater prevalence of cerebrovascular disease, left ventricular dysfunction, diabetes mellitus, chronic obstructive pulmonary disease, renal impairment, and peripheral arterial disease.

This is the reason why the early identification of systemic and cardiovascular comorbidities appear mandatory for the preoperative risk stratification of the elderly candidates to cardiac surgery.

The purpose of this review was to give an outlook on recent developments in cardiac surgery, with the aim of contribution to the decision making and risk reduction in the treatment of elderly patients.

## 2. Age-Related Cardiovascular Changes

Age-related changes occur both in the heart and in small and large vessels, leading to reduced physiological reserves. Most patients show no signs of impaired hemodynamic performance at rest, but surgical and anesthesiological stresses can make their limited cardiac reserve evident [13].

### 2.1. Systemic Vasculature

Aging affects various aspects of vascular morphology and function. The large arteries dilate, and their walls thicken, particularly due to wall matrix changes, increase in elastolytic and collagenolytic activity, and in smooth muscle tone. These modifications lead to increase in vascular stiffness with advancing age [14–16].

In turn, increased vascular stiffness is the main cause of consequent elevated systolic arterial pressure and pulse-wave velocity and early reflected pulse pressure waves and late peak systolic pressure. The final step is the augmentation of aortic impedance and cardiac mechanical load [14]. In this way, arterial stiffening triggers a variety of cardiac adjustments. Some of these adjustments are additional and are similar to the age-related intrinsic changes in cardiac morphology and may, therefore, be expected to worsen cardiac performance [17].

### 2.2. Heart

It has been demonstrated that aging is associated with numerous ionic, molecular, and biochemical changes in the heart [14, 18]. These changes seem to affect protein function, mitochondrial oxidative phosphorylation, excitation-contraction coupling, calcium kinetics, myofilament activation, matrix composition and regeneration, cell growth and size, and apoptosis process [14]. Age-related modifications in cardiac morphology are particularly the result of alterations of intracellular molecular and biochemical pathways. In turn, many of the changes in cardiac function with older age develop as a consequence of underlying alterations in morphology. Finally, cardiac aging results in decreased mechanical and contractile efficiency, stiffening of myocardial cells, mural connective tissue and valves, decreased number of myocytes, increased myocyte size, increased rate of myocyte apoptosis, and blunted-adrenoceptor-mediated contractile and inotropic response [19].

### 2.3. Coronary Circulation

Aging is associated with structural and functional modifications in the coronary vasculature, which could affect myocardial perfusion. The gradual age-related reduction in coronary flow reserve may be a result of abnormal vasodilator capacity or elevated baseline cardiac work and myocardial blood flow [17]. The reduced vasodilator reserve may be the consequence of impaired endothelium-dependent dilation of large epicardial and resistance coronary vessels [20], decreased basal and stimulated release of nitric oxide by the coronary endothelium [21], or increased coronary vasoconstrictor effect of endothelin-1 (ET-1) [22], whose plasma concentrations increase in the elderly [23].

### 2.4. Frailty and Cardiovascular Disease

Chronological age does not always reflect biological status, which may vary in degree of frailty. The concept of “frailty” among older patients with cardiovascular disease has emerged primarily from the geriatrics literature, where frailty is considered a biological state characterized by increased vulnerability and decreased resistance to physiological stresses [24–26]. From a clinical perspective, frailty is associated with loss of skeletal muscle mass, weight loss, reduced walking speed, exhaustion, and reduced activity [24]. Catabolic response to stress is of great concern in the elderly, because of the limited muscle mass of many older people before surgery [27]. Hormonal dysregulation (decline in anabolic hormones) and postoperative systemic inflammation, often through a synergistic interaction, play a fundamental role in the pathogenesis of the so-called geriatric “frailty syndrome” [28]. In recent years, this concept of frailty has been applied to patients presenting with acute cardiac illness as well as those undergoing procedural interventions such as cardiac surgery. Moreover, it has been previously demonstrated that the assessment of frailty and disability in patients undergoing cardiac surgery risk might improve the identification of operative risk [29].

## 3. Aortic Valve Surgery

Aortic stenosis (AS) is the most common heart valve disease in Western countries and occurs rather frequently in elderly patients, since the prevalence of AS is related to age. It has been shown that elderly patients aging 75 years or older benefit from aortic valve replacement (AVR) in comparison to conservatively treated patients, with excellent results [30, 31]. Changes in AVR surgery during the past decade are partly due to growing evidence that the benefits of AVR extend to very old patients [32]. Brown et al. focused on aortic valve replacement without CABG surgery in the society of thoracic surgeons database and reported operative mortality decreasing from 3.5% to 2.4% between 1997 and 2006 [33]. Lee et al. showed that operative mortality for aortic valve replacement decreased from 5.6% during the years 1993–1997 to 4.4% during the years 2003–2007 [34]. There is uncertainty about the benefit of performing coronary artery bypass graft (CABG) surgery during AVR in patients with stable coronary disease, because CABG has been demonstrated to increase perioperative and 1-year mortality. A recent serial cross-sectional cohort study showed that the AVR procedure rate increased by 19 procedures per 100000 person-years over the 12-year period from 1999 to 2011, with an age-, sex-, and race-adjusted rate increase of 1.6% per year. Thirty-day and 1-year mortality was 3.5% and 9.9% in isolated AVR versus 5.1% and 12.3% in AVR with concomitant CABG, respectively [35].

Moreover, in the recent literature, there is a growing body of evidence to consider earlier, preemptive AVR in selected asymptomatic patients [36]. Despite strong recommendations that bioprostheses rather than mechanical valves should be used for patients aging 65 years or older [37, 38], a recent large series from USA [35] reported that 23.9% of patients 85 years old and older continued to receive a mechanical prosthesis in 2011.

However, the decision to operate on elderly patients for symptomatic AS raises specific problems, particularly related to the frequent concomitant comorbidities that can worsen the outcomes of elderly patients suffering from severe aortic stenosis. The Euro Heart Survey reported that nearly one-third of patients with symptomatic severe aortic valve stenosis and age ≥80 were denied standard of care, namely, AVR, and main reasons included advanced age and age-related comorbidities [39]. We recently showed in a large population of elderly patients who undergone AVR that octogenarians' survival rate was similar to the expected survival of the age- and sex-matched regional population and that the clinical conditions at time of surgery (Canadian cardiovascular society class III–IV, preoperative high creatinine levels, extracardiac arteriopathy, and peripheral neurological dysfunction) and not the older age alone emerged as independent risk factors for decreased 6-year survival [31]. Similar results have been found by other authors reporting that in these patients independent predictors of late death included older age, renal failure, diabetes mellitus, stroke, myocardial infarction, immunosuppression, prior CABG, implanted pacemaker, low left ventricular ejection fraction, hypertension, and New York Heart Association class III or IV [38].

Recently, transcatheter aortic valve implantation (TAVI) was demonstrated to provide good clinical and hemodynamic outcomes both in inoperable patients and in high-risk elderly patients [40], and during the last few years the number of procedures, as well as performing centers and performing physicians, has rapidly increased.

A study by Himbert et al. [41] of high-risk patients with AS showed that either transfemoral or transapical aortic valve implantation expands the scope of the treatment of AS in elderly patients and provides satisfactory results in 1 year in this population. D'Onofrio et al. confirmed recently that TAVI provides excellent early and 2-year results in terms of survival, valve-related adverse events, and hemodynamic performance in a series of 179 patients who underwent transapical aortic valve implantation in high-risk patients with severe aortic valve stenosis [42].

However, there is a lack of information on the incidence and predictors of both early mortality at 30 days and late mortality. Recently, Tamburino et al. [43] studied a total of 663 consecutive patients (mean age, 81 years) who underwent TAVI with the third-generation 18-F CoreValve device in 14 centers. Procedural success and intraprocedural mortality were 98% and 0.9%, respectively. The cumulative incidences of mortality were 5.4% at 30 days, 12.2% at 6 months, and 15.0% at 1 year. Clinical and hemodynamic benefits observed acutely after TAVI were sustained at 1 year. Paravalvular leakages were trace to mild in the majority of cases. Conversion to open heart surgery, cardiac tamponade, major access site complications, left ventricular ejection fraction <40%, prior balloon valvuloplasty, and diabetes mellitus were independent predictors of mortality at 30 days, whereas prior stroke, postprocedural paravalvular leak, prior acute pulmonary edema, and chronic kidney disease were independent predictors of mortality between 30 days and 1 year. The data from this study showed that, while early mortality was mainly affected by procedural complications, late mortality was influenced primarily by comorbidities. Importantly, the logistic EuroSCORE showed a weak discriminative ability in predicting 30-day and 1-year mortality, highlighting the need for novel TAVI-specific scores.

## 4. Coronary Revascularization in the Elderly

Myocardial revascularization procedures are becoming more common for people aging ≥80 years, partly because of the favorable outcomes of coronary artery bypass grafting (CABG) and percutaneous coronary interventions (PCI) [44]. CABG has been demonstrated to achieve excellent intermediate survival [45–48], while the use of PCI in these high-risk patients is attractive because of its minimally invasive nature and lower operative mortality [44].

However, the best option in patients aging ≥80 years is largely unknown. Clinical trials frequently exclude elderly patients [49, 50]. Large randomized trials and meta-analyses have examined the appropriateness of each type of intervention in low-risk patients and in patients younger than 80 years [44, 51, 52], but it is unclear whether these data can be extrapolated to older patients. The evidence for the optimal revascularization procedure in octogenarians is therefore based primarily on observational studies, predominantly single center series with a relatively small population size and with an intermediate duration of follow-up [53–55].

Selection of the most appropriate treatment for elderly patients with ischemic heart disease requires careful evaluation on individual basis of the risks and benefits of three approaches: medical therapy, PCI, and CABG surgery. The trial of invasive versus medical therapy in elderly patients with chronic symptomatic coronary artery disease (TIME), a prospective randomized trial involving 305 patients with angina, reported that, after 6 months, revascularization performed both with PCI or CABG was associated with an improved quality of life and fewer major cardiac events (19 versus 49%) compared with medical treatment [56]. The Alberta provincial project for outcome assessment in coronary heart disease (APPROACH) observational study analyzed the absolute risk reduction in late mortality following CABG or PCI compared with medical therapy, based on more than 6000 elderly patients with ischemic heart disease [57]. Interventional approach resulted in improved outcome in all groups compared with medical therapy and the greatest benefit was reported in the oldest patients (>80 years of age), with survival improvements of 17.0% for CABG and 11.3% for PCI, respectively.

### 4.1. Outcomes of PCI in the Elderly

In the elderly, PCI is beneficial, but the rate of complications significantly increases [58, 59] and coronary anatomy is often less amenable [60]. A recent study on PCI in elderly was conducted in 8828 octogenarians during 1998–2000. The authors found angiographic success in 93% with stents placed in 75% of patients [61] and a postprocedural mean length of stay of 3.3 days with an in-hospital mortality of 3.77%, decreasing to 1.35%, in patients without recent myocardial infarction. Over the last 10 years, device technology of drug-eluting stents (DES) has evolved with a concomitant improvement in outcomes of PCI in the elderly. Results from the randomized multicenter SPIRIT III trial comparing the outcomes in elderly and younger patients treated with DES have shown that implantation of these devices appears to be safe in elderly patients [62]. However, currently published experience of the use of DES in the elderly is limited. Furthermore, the long-term outcomes of current PCI technology in the elderly are yet to be determined.

### 4.2. Outcomes of Conventional CABG in the Elderly

It is well demonstrated that CABG in octogenarians relieves angina effectively [63]. The crude survival rates after CABG in individuals aged >75 years in the UK have increased from 92.4% (*n* = 821) in 1998 to 94.1% (*n* = 1804) in 2001 [64]. Moreover, recent literature reports an operative mortality between 2.7 and 6.4% for isolated CABG and a 5-year life expectancy of 65% [65–67]. Wilson et al. studied in-hospital outcomes and cost among 2272 elderly people (>75 years) and 9745 younger patients (<75 years) who underwent CABG between 1997 and 2001 [68]. After controlling for clinical differences, it is demonstrated that patients with age >75 years require a longer length of hospital stay, higher mortality rates and higher in-hospital cost. Recent data from prospective, randomized trials and registry studies reported more favorable outcome with conventional CABG surgery over PCI, especially for diabetes and patients above 65 years of age [67, 69, 70]. Over time, prevalence of postoperative complications and use of hospital resource have significantly reduced in this population. Maganti et al. [66] have reported a regression in operative mortality among octogenarians from 7.1 to 3.2% along with a decreased incidence of postoperative complications such as stroke, low cardiac output, and use of intra-aortic balloon pump. However, they documented an increased prevalence of risk factors such as diabetes, dyslipidemia, hypertension, and left main disease in this population.

Data available and useful to identify those elderly patients who are likely to have an improvement in quality of life after CABG are limited. Although hospital stay length may be longer for elderly patients, psychological and social recovery patterns through the first 6 weeks postoperatively showed to be similar to those of a younger age group [71]. More recently, survivors among octogenarians who underwent isolated CABG reported to have an excellent quality of life for up to 5 years after surgery [72]. Hence, excellent long-term survival after CABG in elderly people may indeed be accompanied by an equally satisfactory quality of life in the majority. Finally, a recent review has been published with the aim to provide an evidence-based overview of the health-related quality of life (HRQOL) data from the literature in elderly patients undergoing CABG [73]. The main findings of this study are that elderly patients have an improved early and late HRQOL following CABG. This issue can allow elderly patients to have HRQOL comparable to an age-matched general population. Derived benefits in HRQOL may be similar in magnitude to younger patients. These results stress the positive impact of CABG on HRQOL of elderly patients. Interestingly, this review should encourage clinicians to evaluate potential CABG surgical patients on the basis of their comorbidities rather than on the basis of age alone as a precluding factor.

### 4.3. On-Pump versus Off-Pump Coronary Surgery in Elderly Patients

Off-pump coronary artery bypass (OPCAB) grafting was recently demonstrated to be an effective surgical technique and may be of potential benefit in high-risk populations as the elderly. The increasing interest in OPCAB is related to the possibility of avoiding the well-known deleterious effects of the CPB pump [74]. In fact, OPCAB is clearly associated with reduction of consequences of systemic inflammatory response syndrome (SIRS) [75]. This syndrome results from a cascade of events generated by the contact of plasma proteases and blood cells with the gaseous interface and bioincompatible surfaces of the CPB tubes. It is well known that the elderly are particularly susceptible to damage by these inflammatory mediators [76]. There is a growing body of evidence that OPCAB is associated with reductions in the risks for stroke, decline of neurocognitive functions, delirium, atrial fibrillation, and acute kidney injury that are the common risks encountered in this population. Moreover, this approach seems to reduce transfusion and inotrope requirements, ventilation time, intensive care unit and hospital stays, and in-hospital and 1-year direct costs [77–79].

Most of published papers reported similar in-hospital mortality rates for conventional CABG and OPCAB in elderly patients [80–83]. However, some recent nonrandomized studies [84] have shown that mortality is higher in the patient group operated with CPB as compared to patients operated without CPB. Another major matter of debate is that CABG is associated with adverse neurological complications. Despite the improvement in surgical and anesthesiological techniques and ECC management, persistent stroke rate associated with CABG has been reported ranging from 1% to 5% of patients [85]. Elderly age has been recognized as an independent predictor of stroke [86], related to the higher prevalence of diseased aorta which is the main cause of perioperative macro and microembolism from aortic arch plaques.

Two meta-analyses of all the observational studies published between 1999 and 2002 [87, 88] showed that the OPCAB technique was associated with significantly lower incidence of stroke in elderly patients compared with the CPB technique. Moreover, off-pump surgery has been shown to better preserve neurocognitive impairment than conventional CABG surgery [89, 90], although the pathophysiology of these neurocognitive deficits appears to be multifactorial.

Two recent meta-analyses have questioned the influence of OPCAB surgery on postoperative atrial fibrillation (AF) [91, 92]. Their results suggested that OPCAB surgery could reduce the incidence of postoperative AF in the general population (age <70 years), but the optimal “protective effect” was not as strong as recorded in the older population.

Renal dysfunction is a serious complication after CABG with cardiopulmonary bypass and it can depend on nonpulsatile flow, hypothermia, hemolysis, systemic inflammatory reactions, and emboli. Moreover, advanced age is an important predictor of postoperative renal dysfunction after CABG [93]. Although a recent propensity-based study on consistent number of patients confirmed these findings [94], a meta-analysis of 6 randomized controlled trials and 16 observational studies failed to show strong benefit in the elderly population regarding OPCAB and renal failure [95].

However, it is significant that, in elderly patients, coronary artery disease is often very severe and characterized by diffuse calcifications compared to the younger population, making off-pump multivessel complete revascularization in these patients very difficult. Moreover, recent studies have confirmed that complete revascularization improves long-term cardiac survival and functional status [96]. In consideration of these findings, a rigorous selection of potential candidates for off-pump technique appears to be mandatory, particularly in the setting of high-risk patients. Even though consensus has been reached on the possibility of performing complete revascularization with the off-pump technique, beating heart coronary surgery for multivessel disease can be performed successfully only by experienced surgeons in high volume centers.

## 5. Mitral Valve Surgery

The decision whether to refer an elderly patient with severe mitral regurgitation for mitral valve operation and the appropriate timing of operative intervention are challenging clinical problems. Mitral valve repair is preferred over replacement because of its well-documented advantages, including lower operative mortality, superior quality of life, higher long-term survival, and greater freedom from reoperation [97–101]. Despite these advantages, mitral valve repair is less frequently performed in the elderly compared with younger patients. According to the society of thoracic surgeons adult cardiac surgery database (STS database), the overall repair rate for isolated primary mitral regurgitation is 60%, whereas the repair rate among patients older than 80 years is 45% [98]. There are several explanations for this disparity: annular/valvular calcification and tissue fragility are more common among elderly patients, there is a general tendency to perform a “quick” mitral valve replacement rather than a complex repair in the elderly, and the evidence of advanced anatomic disease all may discourage aggressive use of repair techniques in these high-risk patients.

No randomized trials are available comparing the outcomes of mitral valve repair with replacement in elderly patients with mitral regurgitation. Then, it remains a matter of debate whether mitral valve repair confers a survival benefit over replacement in the elderly population.

A number of retrospective studies [102–104] have demonstrated the advantages of repair over replacement. Chikwe et al. [102] studied 322 elderly patients who underwent mitral valve repair (70%) or replacement (30%) in two high volume centers. Mean follow-up time was 2.4 years. Mitral valve replacement was associated with significantly higher operative mortality rather than repair (11% for repair versus 19% for replacement). Significant multivariable predictors of operative mortality included concomitant CABG, ejection fraction less than 30%, mitral valve replacement, and emergent surgery. Adjusted survival among patients with degenerative mitral regurgitation was significantly better in patients who had undergone repair than in those with replacement [102].

In contrast, other studies have shown no difference in long-term survival between mitral valve repair and replacement in the elderly population. In one retrospective study, 10-year survival of patients aging 60 years was not statistically higher in patients undergoing mitral valve repair than in those undergoing replacement [105]. Lorusso et al. [106] have recently demonstrated in a similar population that mitral valve replacement is a suitable option for patients with chronic ischemic mitral regurgitation and impaired left ventricular function. It provides better results in terms of freedom from reoperation with comparable valve-related complication rates.

Gillinov et al. [107] studied 3286 patients undergoing isolated primary operation for degenerative mitral valve disease. Valve repair was performed in 93% and replacement was performed in 7% of patients. Patients who underwent valve replacement were older, had more advanced symptoms, and had more complex disease. Among a small group of propensity-matched pairs (6% of the repair population), survival and freedom from mitral valve reoperation were similar between replacement and repair groups. The authors concluded that it is reasonable to perform mitral valve repair in the large majority of elderly patients with mitral regurgitation, although valve replacement does not compromise long-term outcomes when valve disease is complex.

When mitral valve repair is not feasible, bioprostheses are associated with better long-term freedom from valve-related morbidity and excellent freedom from reoperation in elderly patients undergoing mitral valve replacement for mitral regurgitation [108].

There are limited retrospective data available about the optimal timing of mitral surgery in elderly patients. Badhawar et al. [109] have examined the outcomes of mitral valve operation on the basis of preoperative NYHA functional class. They found that operative mortality was significantly higher among patients with advanced heart failure at the time of operation. Five-year survival was 68% among patients with NYHA class III-IV compared with 85% among those with NYHA class I-II. Severe preoperative symptoms were associated with 5-year readmission for congestive heart failure after successful mitral valve repair. These findings indicate that operative intervention should be considered before significant symptoms of heart failure develop in elderly patients with severe mitral regurgitation.

New approaches to mitral valve surgery, in particular various catheter-based techniques, are being tested and seem to be an alternative to open heart surgery for older and sicker patients. These modalities include placing devices into the coronary sinus to support the mitral annulus and direct percutaneous leaflet repair [110, 111]. These approaches are attractive for older patients and could be treatments available to many patients with prohibitive risk with the current options.

## 6. Aortic Surgery

Traditionally, surgery of the ascending aorta and the aortic arch for aortic aneurysms and aortic dissections carries a prohibitive risk for elderly patients. Deep hypothermia, long cardiopulmonary bypass and aortic cross-clamping time, potential need for circulatory arrest, and selective brain perfusion are all risk factors in this kind of surgery. However, recent data show that more and more elderly patients undergo cardiac surgery for diseases of the aorta [112]. Progress in technology and surgical and anesthesiological management led to better outcome of cardiac operations in octogenarians. Chen and Hsu [113] showed that aortic surgery requiring deep hypothermic circulatory arrest can be performed in elderly patients with acceptable results. As a consequence, an increased number of elderly patients are referred for aortic surgery [114].

Meticulous patient selection, excellent brain perfusion, adequate myocardial protection, and a perfect surgical procedure are basic requirements for successful aortic surgery, especially for elderly patients. Recently, alternative surgical techniques in aortic aneurysm surgery, such as debranching of the aortic arch and subsequent aortic endoprosthesis implantation, have gained popularity and offer an option for older and sicker patients. Stenting of the aorta, which was introduced into clinical practice in the mid-1990s, makes treatment of aortic diseases possible even in elderly patients with comorbidities [112].

Nevertheless, these operations remain to be high-risk interventions. The main limitation in the clinical decision making of the management of a patient aging 80 years and older requiring major surgery is often the lack of evidence on the benefits of any intervention. In particular, there are few data on the value of treatment of aortic dissections in these patients. In 2001, Neri et al. [115] reported an experience involving 24 octogenarian patients who underwent open repair of type A aortic dissections with discouraging results. The authors reported an intraoperative mortality rate of 33%, a hospital mortality rate of 83%, and no 6-month survivors and concluded that “older patients should be denied such complicated surgical interventions to conserve resources…”

On the other hand, several groups have published different reports on acute type A aortic dissection repair in octogenarians. Hata et al. [116] reported on a series of 58 octogenarians affected by type A dissections, of whom 30 underwent emergent repair and 28 were managed medically. The early mortality rate was significantly lower in the patients who underwent surgery than in the medically managed group, with 13.3% versus 60.7%, respectively. However, midterm survival was not significantly different between groups, both of which experienced about 40% survival at 8 years.

Piccardo and associates' multicenter report from Europe showed an in-hospital mortality rate of 46% in a series of 57 octogenarians. Similar to the study by Hata, survival beyond 1 year remained reasonable, at about 50% at 5 years [117]. A pooled analysis of type A aortic dissections in the octogenarians was performed by Biancari et al. [118]. The authors reported a 37% early mortality rate and 12% incidence of stroke in 308 octogenarians. On the basis of these results, it seems reasonable that open repair can be considered for most octogenarians although early mortality rate remains high. Unfortunately, few pieces of data exist regarding functional outcome and quality of life of this patient cohort after acute type A aortic dissections repair. More data on the burden of treatments, the intermediate survival, and quality of life of these patients are needed to better establish the role of emergency surgery for major aortic surgery in octogenarians and nonagenarians.

## 7. Conclusions

In summary, we can conclude that, due to technical and medical improvements, cardiac surgery is feasible in elderly patients with acceptable risk in terms of mortality and morbidity. Decision making is rather difficult in elderly patients. Future studies should aim to identify, from the multitude of biological, psychological, and emotional factors, the ones that substantially influence the postoperative result. Flawless surgery, meticulous hemostasis, excellent myocardial protection, and perfect anesthesiological management are basic requirements for cardiac surgery in elderly patients. The care of elderly cardiac surgical patients can be improved only through the strict collaboration of geriatricians, anesthesiologists, cardiologists, and cardiac surgeons, in order to obtain a tailored treatment for the individual patient.

## References

[1] Hannan EL, Kilburn H, Racz M, Shields E, Chassin MR (1994). Improving the outcomes of coronary artery bypass surgery in New York State. *Journal of the American Medical Association*.

[2] Ghali WA, Ash AS, Hall RE, Moskowitz MA (1997). Statewide quality improvement initiatives and mortality after cardiac surgery. *Journal of the American Medical Association*.

[3] Spencer G (1989). *Projections of the Population of the United States, By Age, Sex and Race: 1988–2080. Current Population Reports, Population Estimates and ProjectionsGovernment Printing Office*.

[4] National Center for Health Statistics (1992). *Vital Statistics of the United States US Government Printing Office*.

[5] Friedrich I, Simm A, Kötting J, Thölen F, Fischer B, Silber RE (2009). Cardiac surgery in the elderly patient. *Deutsches Ärzteblatt international*.

[6] Fruitman DS, MacDougall CE, Ross DB (1999). Cardiac surgery in octogenarians: can elderly patients benefit? Quality of life after cardiac surgery. *Annals of Thoracic Surgery*.

[7] Alexander KP, Anstrom KJ, Muhlbaier LH (2000). Outcomes of cardiac surgery in patients age >or = 80 years: results from the national cardiovascular network. *Journal of the American College of Cardiology*.

[8] Trummer G, Beyersdorf F (2005). Heart surgery in the elderly. *Deutsche Medizinische Wochenschrift*.

[9] Shapira I, Pines A, Mohr R (2001). Updated review of the coronary artery bypass grafting option in octogenarians: good tidings. *American Journal of Geriatric Cardiology*.

[10] Hassan A, Newman A, Ko DT (2010). Increasing rates of angioplasty versus bypass surgery in Canada, 1994–2005. *American Heart Journal*.

[11] Lee DH, Buth KJ, Martin B-J, Yip AM, Hirsch GM (2010). Frail patients are at increased risk for mortality and prolonged institutional care after cardiac surgery. *Circulation*.

[12] Sündermann S, Dademasch A, Praetorius J (2011). Comprehensive assessment of frailty for elderly high-risk patients undergoing cardiac surgery. *European Journal of Cardio-thoracic Surgery*.

[13] Loran DB, Zwischenberger JB (2004). Thoracic surgery in the elderly. *Journal of the American College of Surgeons*.

[14] Lakatta EG (1999). Cardiovascular aging research: the next horizons. *Journal of the American Geriatrics Society*.

[15] Lakatta E (1994). Aging effects on the vasculature in health: risk factors for cardiovascular disease. *American Journal of Geriatric Cardiology*.

[16] Priebe H-J (2000). The aged cardiovascular risk patient. *British Journal of Anaesthesia*.

[17] Czernin J, Muller P, Chan S (1993). Influence of age and hemodynamics on myocardial blood flow and flow reserve. *Circulation*.

[18] Folkow B, Svanborg A (1993). Physiology of cardiovascular aging. *Physiological Reviews*.

[19] Olivetti G, Melissari M, Capasso JM, Anversa P (1991). Cardiomyopathy of the aging human heart. Myocyte loss and reactive cellular hypertrophy. *Circulation Research*.

[20] Egashira K, Inou T, Hirooka Y (1993). Effects of age on endothelium-dependent vasodilation of resistance coronary artery by acetylcholine in humans. *Circulation*.

[21] Amrani M, Goodwin AT, Gray CC, Yacoub MH (1996). Ageing is associated with reduced basal and stimulated release of nitric oxide by the coronary endothelium. *Acta Physiologica Scandinavica*.

[22] Goodwin AT, Amrani M, Marchbank AJ, Gray CC, Jayakumar J, Yacoub MH (1999). Coronary vasoconstriction to endothelin-1 increases with age before and after ischaemia and reperfusion. *Cardiovascular Research*.

[23] Miyauchi T, Yanagisawa M, Iida K (1992). Age- and sex-related variation of plasma endothelin-1 concentration in normal and hypertensive subjects. *American Heart Journal*.

[24] Fried LP, Tangen CM, Walston J (2001). Frailty in older adults: evidence for a phenotype. *Journals of Gerontology A Biological Sciences and Medical Sciences*.

[25] Boyd CM, Xue Q-L, Simpson CF, Guralnik JM, Fried LP (2005). Frailty, hospitalization, and progression of disability in a cohort of disabled older women. *American Journal of Medicine*.

[26] Rockwood K, Stadnyk K, MacKnight C, McDowell I, Hebert R, Hogan DB (1999). A brief clinical instrument to classify frailty in elderly people. *The Lancet*.

[27] Wolfe RR (2006). Optimal nutrition, exercise, and hormonal therapy promote muscle anabolism in the elderly. *Journal of the American College of Surgeons*.

[28] Fried L, Walston J, Hazzard WR, Blass JP, Ettinger WH, Halter JB, Ous-lander J (2003). Frailty and failure to thrive. *Principles of Geriatric Gerontology*.

[29] Afilalo J, Mottillo S, Eisenberg MJ (2012). Addition of frailty and disability to cardiac surgery risk scores identifies elderly patients at high risk of mortality or major morbidity. *Cardiovascular Quality and Outcomes*.

[30] Mortasawi A, Gehle S, Yaghmaie M (2001). Early and long-term results of aortic valve replacement in patients aged 80 years and older. *Herz*.

[31] Di Eusanio M, Fortuna D, Cristell D (2012). Contemporary outcomes of conventional aortic valve replacement in 638 octogenarians: insights from an Italian Regional Cardiac Surgery Registry (RERIC). *European Journal Cardio-Thoracic Surgery*.

[32] Cohn LH, Narayanasamy N (2007). Aortic valve replacement in elderly patients: what are the limits?. *Current Opinion in Cardiology*.

[33] Brown JM, O’Brien SM, Wu C, Sikora JAH, Griffith BP, Gammie JS (2009). Isolated aortic valve replacement in North America comprising 108,687 patients in 10 years: changes in risks, valve types, and outcomes in the Society of Thoracic Surgeons National Database. *Journal of Thoracic and Cardiovascular Surgery*.

[34] Lee R, Li S, Rankin JS (2011). Fifteen-year outcome trends for valve surgery in North America. *Annals of Thoracic Surgery*.

[35] Barreto-Filho JA, Wang Y, Dodson JA (2013). Trends in aortic valve replacement for elderly patients in the United States 1999–2011. *The Journal of the American Medical Association*.

[36] Vaishnava P, Fuster V, Goldman M, Bonow RO (2011). Surgery for asymptomatic degenerative aortic and mitral valve disease. *Nature Reviews Cardiology*.

[37] Bonow RO, Carabello BA, Chatterjee K (2008). 2008 Focused update incorporated into the ACC/AHA 2006 guidelines for the management of patients with valvular heart disease: a report of the American College of Cardiology/American Heart Association Task Force on Practice Guidelines (Writing Committee to Revise the 1998 Guidelines for the Management of Patients With Valvular Heart Disease): endorsed by the Society of Cardiovascular. *Circulation*.

[38] Ashikhmina EA, Schaff HV, Dearani JA (2011). Aortic valve replacement in the elderly: determinants of late outcome. *Circulation*.

[39] Iung B, Baron G, Butchart EG (2003). A prospective survey of patients with valvular heart disease in Europe: the Euro Heart Survey on valvular heart disease. *European Heart Journal*.

[40] Smith CR, Leon MB, Mack MJ (2011). Transcatheter versus surgical aortic-valve replacement in high-risk patients. *The New England Journal of Medicine*.

[41] Himbert D, Descoutures F, Al-Attar N (2009). Results of transfemoral or transapical aortic valve implantation following a uniform assessment in high-risk patients with aortic stenosis. *Journal of the American College of Cardiology*.

[42] D’Onofrio A, Fusari M, Abbiate N (2011). Transapical aortic valve implantation in high-risk patients with severe aortic valve stenosis. *Annals of Thoracic Surgery*.

[43] Tamburino C, Capodanno D, Ramondo A (2011). Incidence and predictors of early and late mortality after transcatheter aortic valve implantation in 663 patients with severe aortic stenosis. *Circulation*.

[44] McKellar SH, Brown ML, Frye RL, Schaff HV, Sundt TM (2008). Comparison of coronary revascularization procedures in octogenarians: a systematic review and meta-analysis. *Nature Clinical Practice Cardiovascular Medicine*.

[45] Nissinen J, Wistbacka J-O, Loponen P (2010). Coronary artery bypass surgery in octogenarians: long-term outcome can be better than expected. *Annals of Thoracic Surgery*.

[46] Filsoufi F, Rahmanian PB, Castillo JG, Chikwe J, Silvay G, Adams DH (2007). Results and predictors of early and late outcomes of coronary artery bypass graft surgery in octogenarians. *Journal of Cardiothoracic and Vascular Anesthesia*.

[47] Baskett R, Buth K, Ghali W (2005). Outcomes in octogenarians undergoing coronary artery bypass grafting. *Canadian Medical Association Journal*.

[48] Nicolini F, Molardi A, Verdichizzo D (2012). Coronary artery surgery in octogenarians: evolving strategies for the improvement in early and late results. *Heart and Vessels*.

[49] Mullany CJ, Darling GE, Pluth JR (1990). Early and late results after isolated coronary artery bypass surgery in 159 patients aged 80 years and older. *Circulation*.

[50] Singh M, Mathew V, Garratt KN (2000). Effect of age on the outcome of angioplasty for acute myocardial infarction among patients treated at the Mayo Clinic. *American Journal of Medicine*.

[51] Rogers WJ, Alderman EL, Chaitman BR (1995). Bypass Angioplasty Revascularization Investigation (BARI): baseline clinical and angiographic data. *American Journal of Cardiology*.

[52] Hoffman SN, TenBrook JA, Wolf MP, Pauker SG, Salem DN, Wong JB (2003). A meta-analysis of randomized controlled trials comparing coronary artery bypass graft with percutaneous transluminal coronary angioplasty: one- to eight-year outcomes. *Journal of the American College of Cardiology*.

[53] Gunn J, Kuttila K, Vasques F (2012). Comparison of results of coronary artery bypass grafting versus percutaneous coronary intervention in octogenarians. *American Journal of Cardiology*.

[54] Wongcharoenkiat N, Tresukosol D, Laksanabunsong P, Udompunturak S (2012). A comparison of outcomes between percutaneous coronary intervention versus coronary artery bypass surgery in octogenarian patients. *Journal of the Medical Association of Thailand*.

[55] Ben-Gal Y, Finkelstein A, Banai S (2012). Surgical myocardial revascularization versus percutaneous coronary intervention with drug-eluting stents in octogenarian patients. *The Heart Surgery Forum*.

[56] Pfisterer M (2001). Trial of invasive versus medical therapy in elderly patients with chronic symptomatic coronary-artery disease (TIME): a randomised trial. *The Lancet*.

[57] Graham MM, Ghali WA, Faris PD, Galbraith PD, Norris CM, Knudtson ML (2002). Survival after coronary revascularization in the elderly. *Circulation*.

[58] Batchelor WB, Anstrom KJ, Muhlbaier LH (2000). Contemporary outcome trends in the elderly undergoing percutaneous coronary interventions: results in 7,472 octogenarians. *Journal of the American College of Cardiology*.

[59] De Gregorio J, Kobayashi Y, Albiero R (1998). Coronary artery stenting in the elderly: short-term outcome and long- term angiographic and clinical follow-up. *Journal of the American College of Cardiology*.

[60] Kowalchuk GJ, Siu SC, Lewis SM (1990). Coronary artery disease in the octogenarian: angiographic spectrum and suitability for revascularization. *American Journal of Cardiology*.

[61] Klein LW, Block P, Brindis RG (2002). Percutaneous coronary interventions in octogenarians in the American College of Cardiology-National Cardiovascular Data Registry: development of a nomogram predictive of in-hospital mortality. *Journal of the American College of Cardiology*.

[62] Hermiller JB, Nikolsky E, Lansky AJ (2011). Clinical and angiographic outcomes of elderly patients treated with everolimus-eluting versus paclitaxel-eluting stents: three-year results from the SPIRIT III randomised trial. *EuroIntervention*.

[63] Hedeshian MH, Namour N, Dziadik E, Stewart RD, Campos CT (2002). Does increasing age have a negative impact on six-month functional outcome after coronary artery bypass?. *Surgery*.

[64] Natarajan A, Samadian S, Clark S (2007). Coronary artery bypass surgery in elderly people. *Postgraduate Medical Journal*.

[65] Zingone B, Gatti G, Rauber E (2009). Early and late outcomes of cardiac surgery in octogenerians. *Annals of Thoracic Surgery*.

[66] Maganti M, Rao V, Brister S, Ivanov J (2009). Decreasing mortality for coronary artery bypass surgery in octogenarians. *Canadian Journal of Cardiology*.

[67] Hlatky MA, Boothroyd DB, Bravata DM (2009). Coronary artery bypass surgery compared with percutaneous coronary interventions for multivessel disease: a collaborative analysis of individual patient data from ten randomised trials. *The Lancet*.

[68] Wilson MF, Baig MK, Ashraf H (2005). Quality of life in octagenarians after coronary artery bypass grafting. *American Journal of Cardiology*.

[69] Dacey LJ, Likosky DS, Ryan TJ (2007). Long-term survival after surgery versus percutaneous intervention in octogenarians with multivessel coronary disease. *Annals of Thoracic Surgery*.

[70] Contini GA, Nicolini F, Fortuna D (2013). Five-year outcomes of surgical or percutaneous myocardial revascularization in diabetic patients. *International Journal of Cardiology*.

[71] Artinian NT, Duggan C, Miller P (1993). Age differences in patient recovery patterns following coronary artery bypass surgery. *American Journal of Critical Care: An Official Publication, American Association of Critical-Care Nurses*.

[72] Rumsfeld JS, Magid DJ, O’Brien M (2001). Changes in health-related quality of life following coronary artery bypass graft surgery. *Annals of Thoracic Surgery*.

[73] Shan L, Saxena A, McMahon R, Newcomb A (2013). Coronary artery bypass graft surgery in the elderly. A review of postoperative quality of life. *Circulation*.

[74] Raja SG, Dreyfus GD (2006). Impact of off-pump coronary artery bypass surgery on postoperative renal dysfunction: current best available evidence. *Nephrology*.

[75] Raja SG, Berg GA (2007). Impact of off-pump coronary artery bypass surgery on systemic inflammation: current best available evidence. *Journal of Cardiac Surgery*.

[76] Marti L, Cervera C, Filella X, Marin JL, Almela M, Moreno A (2007). Cytokine-release patterns in elderly patients with systemic inflammatory response syndrome. *Gerontology*.

[77] Parolari A, Alamanni F, Cannata A (2003). Off-pump versus on-pump coronary artery bypass: meta-analysis of currently available randomized trials. *Annals of Thoracic Surgery*.

[78] Reston JT, Tregear SJ, Turkelson CM (2003). Meta-analysis of short-term and mid-term outcomes following off-pump coronary artery bypass grafting. *Annals of Thoracic Surgery*.

[79] Raja SG, Dreyfus GD (2006). Impact of off-pump coronary artery bypass surgery on postoperative renal dysfunction: current best available evidence. *Nephrology*.

[80] Saleh HZ, Shaw M, Fabri BM, Chalmers JAC (2011). Does avoidance of cardiopulmonary bypass confer any benefits in octogenarians undergoing coronary surgery?. *Interactive Cardiovascular and Thoracic Surgery*.

[81] LaPar DJ, Bhamidipati CM, Reece TB, Cleveland JC, Kron IL, Ailawadi G (2011). Is off-pump coronary artery bypass grafting superior to conventional bypass in octogenarians?. *Journal of Thoracic and Cardiovascular Surgery*.

[82] Jensen BØ, Rasmussen LS, Steinbrüchel DA (2008). Cognitive outcomes in elderly high-risk patients 1 year after off-pump versus on-pump coronary artery bypass grafting. A randomized trial. *European Journal of Cardio-thoracic Surgery*.

[83] Tugtekin S, Kappert U, Alexiou K, Wilbring M, Nagpal AD, Matschke K (2007). Coronary artery bypass grafting in octogenarians—outcome with and without extracorporeal circulation. *Thoracic and Cardiovascular Surgeon*.

[84] Meco M, Biraghi T, Panisi P, Casselman F, Cosseta D, Cirri S (2007). Aortocoronary bypass grafting in high-risk patients over 75 years. Propensity score analysis of on versus off-pump early and midterm results. *Journal of Cardiovascular Surgery*.

[85] Stamou SC, Dangas G, Dullum MKC (2000). Beating heart surgery in octogenarians: perioperative outcome and comparison with younger age groups. *Annals of Thoracic Surgery*.

[86] Puskas JD, Winston AD, Wright CE (2000). Stroke after coronary artery operation: incidence, correlates, outcome, and cost. *Annals of Thoracic Surgery*.

[87] Athanasiou T, Al-Ruzzeh S, Kumar P (2004). Off-pump myocardial revascularization is associated with less incidence of stroke in elderly patients. *Annals of Thoracic Surgery*.

[88] Panesar SS, Athanasiou T, Nair S (2006). Early outcomes in the elderly: a meta-analysis of 4921 patients undergoing coronary artery bypass grafting—Comparison between off-pump and on-pump techniques. *Heart*.

[89] Zamvar V, Williams D, Hall J (2002). Assessment of neurocognitive impairment after off-pump and on-pump techniques for coronary artery bypass graft surgery: prospective randomised controlled trial. *British Medical Journal*.

[90] Lee JD, Lee SJ, Tsushima WT (2003). Benefits of off-pump bypass on neurologic and clinical morbidity: a prospective randomized trial. *Annals of Thoracic Surgery*.

[91] Athanasiou T, Aziz O, Mangoush O (2004). Do off-pump techniques reduce the incidence of postoperative atrial fibrillation in elderly patients undergoing coronary artery bypass grafting?. *Annals of Thoracic Surgery*.

[92] Athanasiou T, Aziz O, Mangoush O (2004). Does off-pump coronary artery bypass reduce the incidence of post-operative atrial fibrillation? A question revisited. *European Journal of Cardio-thoracic Surgery*.

[93] Maitra G, Ahmed A, Rudra A (2009). Renal dysfunction after off-pump coronary artery bypass surgery- risk factors and preventive strategies. *Indian Journal of Anaesthesia*.

[94] Weerasinghe A, Athanasiou T, Al-Ruzzeh S (2005). Functional renal outcome in on-pump and off-pump coronary revascularization: a propensity-based analysis. *Annals of Thoracic Surgery*.

[95] Nigwekar SU, Kandula P, Hix JK, Thakar CV (2009). Off-pump coronary artery bypass surgery and acute kidney injury: a meta-analysis of randomized and observational studies. *American Journal of Kidney Diseases*.

[96] Kleisli T, Cheng W, Jacobs MJ (2005). In the current era, complete revascularization improves survival after coronary artery bypass surgery. *Journal of Thoracic and Cardiovascular Surgery*.

[97] Bonow RO, Carabello BA, Chatterjee K (2008). 2008 focused update incorporated into the ACC/AHA 2006 guidelines for the management of patients with valvular heart disease: a report of the American College of Cardiology/American Heart Association Task Force on Practice Guidelines (writing committee to revise the 1998 guidelines for the management of patients with valvular heart disease). *Circulation*.

[98] Gammie JS, Sheng S, Griffith BP (2009). Trends in mitral valve surgery in the United States: results from The Society of Thoracic Surgeons Adult Cardiac Database. *Annals of Thoracic Surgery*.

[99] Alfieri O, Maisano F (2011). Mitral valve surgery in the elderly: new insights and unanswered questions. *European Heart Journal*.

[100] Sutton MG, Gorman RC (2006). Surgery for asymptomatic severe mitral regurgitation in the elderly: Early surgery or wait and watch?. *Circulation*.

[101] Rosenhek R, Rader F, Klaar U (2006). Outcome of watchful waiting in asymptomatic severe mitral regurgitation. *Circulation*.

[102] Chikwe J, Goldstone AB, Passage J (2011). A propensity score-adjusted retrospective comparison of early and mid-term results of mitral valve repair versus replacement in octogenarians. *European Heart Journal*.

[103] Ailawadi G, Swenson BR, Girotti ME (2008). Is mitral valve repair superior to replacement in elderly patients?. *Annals of Thoracic Surgery*.

[104] Daneshmand MA, Milano CA, Rankin JS (2010). Influence of patient age on procedural selection in mitral valve surgery. *Annals of Thoracic Surgery*.

[105] Thourani VH, Weintraub WS, Guyton RA (2003). Outcomes and long-term survival for patients undergoing mitral valve repair versus replacement: effect of age and concomitant coronary artery bypass grafting. *Circulation*.

[106] Lorusso R, Gelsomino S, Vizzardi E (2013). Mitral valve repair or replacement for ischemic mitral regurgitation? The Italian Study on the Treatment of Ischemic Mitral Regurgitation (ISTIMIR). *The Journal of Thoracic and Cardiovascular Surgery*.

[107] Gillinov AM, Blackstone EH, Nowicki ER (2008). Valve repair versus valve replacement for degenerative mitral valve disease. *Journal of Thoracic and Cardiovascular Surgery*.

[108] Chikwe J, Filsoufi F (2011). Durability of tissue valves. *Seminars in Thoracic and Cardiovascular Surgery*.

[109] Badhawar V, Peterson ED, Jacob JP (2012). Longitudinal outcome of isolated mitral repair in older patients: results from 14,604 procedures performed from 1991 to 2007. *The Annals of Thoracic Surgery*.

[110] Feldman T (2006). Proceedings of TCT: current status of catheter-based mitral valve repair therapies. *Journal of Interventional Cardiology*.

[111] Maisano F, Buzzatti N, Taramasso M, Alfieri O (2013). Mitral transcatheter technologies. *Rambam Maimonides Medical Journal*.

[112] Tsai TT, Trimarchi S, Nienaber CA (2009). Acute aortic dissection: perspectives from the International Registry of Acute Aortic Dissection (IRAD). *European Journal of Vascular and Endovascular Surgery*.

[113] Chen Y-C, Hsu R-B (2008). Aortic surgery requiring hypothermic circulatory arrest in octogenarians. *Journal of the Formosan Medical Association*.

[114] Apostolakis E, Koletsis EN, Dedeilias P (2008). Antegrade versus retrograde cerebral perfusion in relation to postoperative complications following aortic arch surgery for acute aortic dissection type A. *Journal of Cardiac Surgery*.

[115] Neri E, Toscano T, Massetti M (2001). Operation for acute type A aortic dissection in octogenarians: is it justified?. *Journal of Thoracic and Cardiovascular Surgery*.

[116] Hata M, Sezai A, Niino T (2008). Should emergency surgical intervention be performed for an octogenarian with type A acute aortic dissection?. *Journal of Thoracic and Cardiovascular Surgery*.

[117] Piccardo A, Regesta T, Zannis K (2009). Outcomes after surgical treatment for type A acute aortic dissection in octogenarians: a multicenter study. *Annals of Thoracic Surgery*.

[118] Biancari F, Vasques F, Benenati V, Juvonen T (2011). Contemporary results after surgical repair of type A aortic dissection in patients aged 80 years and older: a systematic review and meta-analysis. *European Journal of Cardio-thoracic Surgery*.

